# Methylation mediated Gadd45β enhanced the chemosensitivity of hepatocellular carcinoma by inhibiting the stemness of liver cancer cells

**DOI:** 10.1186/s13578-017-0189-8

**Published:** 2017-12-06

**Authors:** Xiao-Juan Hou, Qiu-Dong Zhao, Ying-Ying Jing, Zhi-Peng Han, Xue Yang, Li-Xin Wei, Yu-Ting Zheng, Feng Xie, Bai-He Zhang

**Affiliations:** 1 Department of Tumor Immunology and Gene Therapy Center, Shanghai Eastern Hepatobiliary Surgery Hospital, NO. 225 Changhai Road, Shanghai, 200438 China; 2 Department of Biliary Tract Surgery Department III, Shanghai Eastern Hepatobiliary Surgery Hospital, Shanghai, China

**Keywords:** Hepatocellular carcinoma, Gadd45β methylation, Apoptosis, Stemness

## Abstract

**Background:**

Defects of the growth arrest DNA damage-inducible gene 45β (Gadd45β) play an important role in the progression of tumor and confer resistance to chemotherapy. However, the role of Gadd45β in the apoptosis of hepatocellular carcinoma is still not clear. Purpose of this study was to explore the effect of Gadd45β on the apoptosis of liver cancer cells, and the possible mechanism was examined.

**Result:**

In this study, we first confirmed the decreased expression of Gadd45β in human liver cancer tissues and human liver cancer cell lines, when compared to the peri-tumor liver tissue and normal liver cells. And, it was found that Gadd45β could inhibit the stemness of liver cancer cells, enhancing the apoptosis of cancer cells induced by chemotherapy. Furthermore, the results showed that HCC tissues and cell lines showed a higher methylation status in Gadd45β promoter than that in peri-tumor tissues and normal liver cells. Methylation was then reversed by pretreatment of SMMC-7721 and Hep-3B with 5-azacytidine which is the DNA methyltransferase inhibitor. And the 5-azacytidine decreased the stemness of SMMC-7721 and Hep-3B, enhanced the sensitivity of SMMC-7721 and Hep-3B to cisplatin.

**Conclusions:**

Methylation mediated Gadd45β expression inhibited the stemness of liver cancer cells, promoting the chemotherapy-induced apoptosis. Thus Gadd45β may be the potential target for enhancing the chemosensitivity of human hepatocellular carcinoma.

## Background

Hepatocellular carcinoma (HCC) is the second leading cause of cancer-related deaths. There are approximately 750,000 new cases of liver cancer each year worldwide [1]. Since the symptom of early-stage HCC is not obvious, hepatocellular carcinoma is often diagnosed at intermediate or advanced stages, when only chemoembolization and sorafenib have shown benefits [2]. However, HCC has a very poor survival rate and prognosis. Thus, it is nessecery to find an effective chemotherapy target for the treatment of HCC.

The Growth Arrest and DNA damage-inducible 45 (Gadd45) gene family contains three members, Gadd45α, Gadd45β, and Gadd45γ. Gadd45 protein are mainly located within the cell nucleus. They are a group of stress-induced protein family, a variety of stresses stimulation can induce their expression, such as UV-radiation, ionizing irradiation, medium starvation, and alkylating agent [3]. The Gadd45 proteins play important roles in the multiple cellular processes, including cell cycle control, proapoptosis, DNA repair [4–7]. These are important event for cellular defense against DNA damage and tumorigenesis. Therefore, the deficiency of Gadd45 gene may contribute to the tumorigenesis. It was found that Gadd45α-deficient mice increased cancer susceptibility [8] [9], and it was also observed that Gadd45 was frequently deficient in tumor cells [10, 11]. In addition, the study indicated that the induction of Gadd45β promoted the therapeutic drug induced apoptosis in hepatocellular carcinoma [12]. However, the mechanism is still not clear.

The role of Gadd45 in apoptosis is complicated in tumor cells. Gadd45 proteins were originally reported as a pro-apoptotic protein; but recent studies showed that Gadd45α and Gadd45β increased tumor cell survival under treatment with chemotherapeutic drugs [13]. Gadd45β proteins are frequently particularly decreased expression in hepatocellular carcinoma (HCC) [10, 14, 15]. However, the role of Gadd45β in the apoptosis of hepatocellular carcinoma is still not clear. In current study, we found that the overexpression of Gadd45β enhanced the chemotherapy induced apoptosis in hepatocellular carcinoma. And the mechanism may depend on the inhibition of stemness of liver cancer cells induced by induction of Gadd45β.

## Methods

### Cell culture, transfection, and drug treatment

Human HCC cell line SMMC-7721 and Hep-3B, and normal liver cell line L-02 were acquired from the Chinese Academy of Sciences Cell Bank. L-02 cells were cultured in 1640 medium, SMMC-7721 and Hep-3B cells were cultured in high glucose Dulbecco’s modified Eagle’s medium with 10% fetal bovine serum, 100 unites/ml of penicillin, and 100 μg/ml of streptomycin (all from Invitrogen) in a humidified atmosphere of 5% CO_2_ at 37 °C.

For overexpression of Gadd45β, Gadd45β-expressing adenovirus were transfected into SMMC-7721 and Hep3B cells according to the manufacturer’s instructions. Briefly, both cells were seeded (2 × 10^5^ cells/well) into 12-well plates overnight. Gadd45β expressing lentivirus were then transfected into the cells. After 24 h of transfections, fresh medium was changed to remove virus. Seventy-two hours after the transfection, the effect of viral on cell were analyzed. Two weeks after transfection, Hep3B^+Gadd45β^ and SMMC-7721^+Gadd45β^ cells were collected by flow cytometry sorting.

5-Azacytidine was purchased from Sigma-Aldrich. SMMC-7721 and Hep-3B cells were pretreated with 10 μm of 5-Azacytidine for 72 h. For determining the sensitivity to cisplatin, after pretreatment of 5-Azacytidine for 72 h, SMMC-7721 and Hep-3B cells were seeded for 24 h and treated with cisplatin (2 μg/ml).

### Patients and specimens

100 cases of HCC and peri-tumor tissues were acquired from Shanghai Eastern Hepatobiliary Surgery Hospital. Informed consent was obtained from each patient under a protocol approved by the Hospital Research Ethics Committees. The patients’ demographics and baseline characteristics are summarized in Table 1. Tumor cell differentiation was assessed basing on the H&E staining as previous describe [10]. Briefly, well differentiated HCC was characterized with minimal atypia and an increased nuclear/cytoplasmic ratio; moderately differentiated HCC were arranged in trabeculae of more cells in thickness and possessed abundant eosinophilic cytoplasma and round nuclei; poorly differentiated HCC had an increased nuclear/cytoplasmic ratio and frequent pleomorphism and bizarre giant cells.

**Table 1:** Relationship between patient demographic features, HCC differentiation and Gadd45β expression (n = 100)

Feature	n = 100	No. expression		P
		Gadd45β (0)	Gadd45β (+ to +++)	
Age (years)				0.18
< 65	28	20	8	
≥ 65	72	60	12	
Gender				0.66
Male	65	40	25	
Female	35	20	15	
Hepatitis B				0.58
Positive	70	55	15	
Negative	30	25	5	
Differentiation				0.009
Well	21	10	11	
Moderate	17	12	5	
Poor	62	58	4	

P < 0.05 was considered to be statistically significant

### Immunohistochemistry

Tumor sections were cut and fixed in 4% formaldehyde solution. Endogenous peroxidase activity was blocked by methanol-3% H_2_O_2_. The primary rabbit anti-human polyclonal Gadd45β antibody (dilution 1:100; Abcam, Cambridge, UK) was used to incubate the tissue sections in a humid container at 4 °C for overnight, goat anti-rabbit antibody (dilution 1:200; Invitrogen, Carlsbad, CA, USA) was used as secondary antibody, and the sections were washed with PBS. Granular cytoplasmic stain with yellow color was considered as positive, no staining was considered as negative when observed under a Leica DMRA microscope (Leica Microsystems Imaging Solutions, Ltd., Cambridge, UK). Furthermore, the positive staining of Gadd45β was classified: + represents less than 30% staining; ++ represents 30–70% staining; +++ represents more than 70% staining.

### Real-time PCR and reverse transcription PCR

To further validate the results of IHC study, Real-Time PCR were performed to examine the Gadd45β expression in fresh liver cancer tissue and surrounding non-neoplastic liver tissue. The selected tissues were first performed the RNA isolation by DNeasy Blood & Tissue Kit (QIAGEN, Hilden, Germany). The cDNA was reverse-transcribed from total RNA using bestar qPCR RT kit (DBI^®^ Bioscience, Germany). The real-time RT-PCR was then carried out by using the SYBR Green Master Mix Kit (DBI^®^ Bioscience, Germany). To examine the Gadd45β expression in liver cancer cells and normal liver cells. The reverse transcription PCR was performed by using 2 × Taq PCR Master Mix (DBI^®^ Bioscience, Germany). In order to normalize the Gadd45β mRNA expression, the endogenous GAPDH mRNA was used. Primers for GAPDH determination were: 5′-AC AACTTTGGTATCGTGGAAGG-3′(forward), 5′-GCCATCACGCCACAGTTTC-3′(reverse); Primers for Gadd45β determination were: 5′-ACGAGTCGGCCAAGTTGATG-3′(forward), 5′-GGATGAGCGTGAAGTGGATTT-3′ (reverse). In addition, to examine the stem character of cells, the following primers were used. Primers for SOX9 determination were: 5′-AGCGAACGCACATCAAGAC–3′(forward), 5′-CTGTAGGCGATCTGTTGGGG-3′(reverse); Primers for OCT4 determination were:5′-CTGGGTTGATCCTCGGACCT-3′(forward), 5′-CCATCGGAGTTGCTCTCCA-3′(reverse). The PCR conditions were set as following: an initial denaturation at 95 °C for 2 min, followed by 40 cycles of 95 °C for 15 s, 60 °C for 60 s, 72 °C for 1 min.

### Western-blot analysis

The expression levels of Gadd45β protein in cells and tissues were assessed by western blot assay. In detail, the cell and tissue samples were firstly collected, and then lysed. The protein concentration of the whole lysates was quantified by the BCA Protein Assay Kit (Pierce, Rockford, IL, USA). The following antibodies were used in the experiment: rabbit anti-human polyclonal antibody to Gadd45β (dilution 1:2000; Abcam, Cambridge, UK), rabbit anti-GAPDH antibody (dilution 1:1000; Abcam); the secondary goat anti-rabbit antibody (dilution 1:5000; Abcam). In addition, to examine the stem character of cells, the used antibodies were specific for SOX9, OCT4, GAPDH (Abcam), and goat anti-rabbit secondary antibody (Abcam).

### Colony formation assay

The transfected and non-transfected SMMC-7721, Hep3B (1 × 10^3^) were seeded in plates. The cells were incubated for 2 weeks to allow the development of colonies. Cell culture medium were moved, and the exposed cells were fixed in ethanol and stained with 2% crystal violet. Then the colony formation of transfected and non-transfected cells can be observed and counted.

### Methylation analysis

To determine whether the decreased expression of Gadd45β is associated with hypermethylation in promoter, methylation-specific PCR (MSP) assay was used to measure the hypermethylation status of Gadd45β promoter as previously described [16]. Before MSP assay, the DNA samples were pre-treated, genomic DNA was isolated by DNeasy Blood & Tissue Kit (QIAGEN, Hilden, Germany) and treated with bisulfate modification using DNA modification kit (QIAGEN, Hilden, Germany). Then bisulfate-treated DNA was used to the MSP amplification using the methylated and un-methylated Gadd45β primers as previous study [17]. The primers for amplification of methylated DNA were 5′-TTCGAAAGTTCGGGTCGTTTCGCGC-3′(forward) and 5′-GGGGACCGAATAAATAACCGCG-3′(reverse); the primer for amplification of unmethylated DNA were: 5′-AAAGTTTGGGTTGTTTTGTGT-3′(forward) and 5′-ACCAAATAAATAACCACA-3′(reverse). PCR products were 137 and 128-bp in the methylated specific and unmethylated primers system, respectively.

### Flow cytometric analysis

The cell apoptosis was measured by an Annexin-APC assay via flow cytometry according to the manufacturer’s instructions (Nanjing Keygen Biotech, China, Cat.KGA108). Briefly, SMMC-7721, Hep3B cells treated with 5-AZA and nontreatment were seeded in 6-well plates to 70–80% cell confluence. The cells were treated with cisplatin for 10 h. Then the cells were collected by trypsinization, washed twice with phosphate-buffered saline (PBS) and resuspended in 200 μl of binding buffer containing 2 μl Annexin and 2 μl PI, incubated for 15 min at room temperature in the dark. After the incubation, at least 10^5^ cells were measured with flow cytometry.

### Statistical analysis

Statistical analysis of data was performed by Student’s *t* test. Data are presented as mean ± SD. Differences between categorical variables were assessed by the Chi square test. P < 0.05 was considered to be statistically significant.

## Results

### Down-regulation of Gadd45β expression in HCC and patient demographic features

**Fig. 1:**
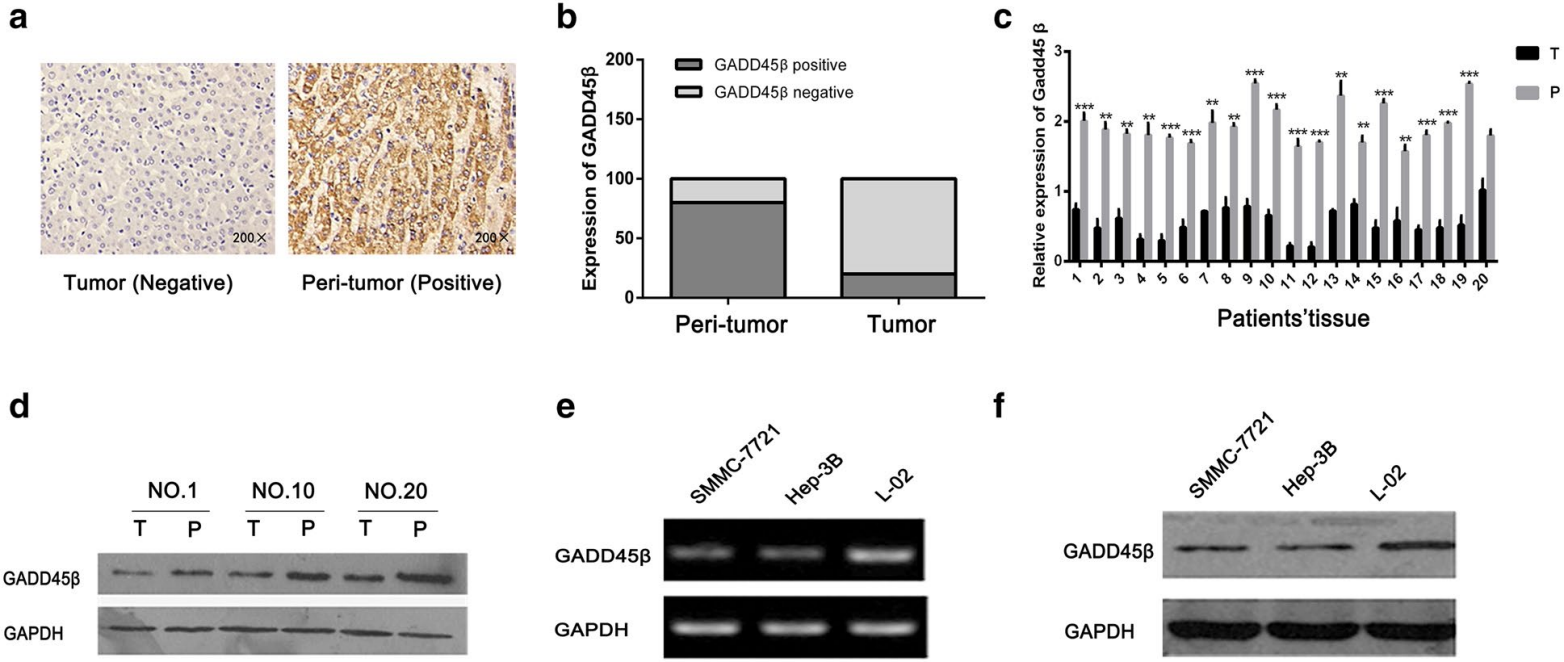
Down-regulation of Gadd45β expression in liver cancer tissues and liver cancer cell lines. **a** The typical IHC images from tumor and peri-tumor tissues with Gadd45β staining were obtained. The tumor tissues showed negative staining. The peri-tumor tissues indicated the obviously positive staining. **b** The IHC staining of 100 paired tissue samples was classified. The peri-tumor tissues showed the 80% positive staining; the tumor tissues indicated only 20% positive staining. **c** The Gadd45β mRNA expression in 20 paired of fresh tissue samples was examined by RT-PCR assay. The tumor tissues showed the relatively lower Gadd45β expression, compared to peri-tumor tissues. GAPDH was used as an internal reference. (**P < 0.01; ***P < 0.001). **d** Western blot analysis showed the lower Gadd45β protein level in tumor tissues, compared to peri-tumor tissues. The protein levels were normalized by a comparison with the GAPDH level. **e**, **f** Gadd45β expression in HCC cell lines (SMMC-7721, Hep-3B) and normal liver cells (L-02) was examined by PCR (**e**) and western blot (**f**), GAPDH was used as an internal reference

We firstly examined Gadd45β expression in one hundred of HCC cases and peri-tumor tissues by immunohistochemistry (IHC) staining. Figure 1a showed the typical image of negative and positive staining of Gadd45β in tumor and peri-tumor tissues, respectively. Furthermore, according to the staining pattern of Gadd45β as described aboved, we found that the peri-tumor tissue showed 80% (80 of 100) positive staining, while tumor tissue showed only 20% (20 of 100) positive staining (Fig. 1b). To validate the IHC analyses, 20 paired of fresh HCC and peri-tumor samples were further examined by the RT-PCR study. The quantitative analysis showed that Gadd45β mRNA level in liver cancer tissue were readily lower than that in peri-tumor tissues (**P < 0.01; ***P < 0.001) (Fig. 1c). Furtherly, western blot results also showed the down-regulation of Gadd45β expression in tumor tissues, when compared to the peri-tumor tissues (Fig. 1d).

To further confirm the difference of Gadd45β expression in tumor and peri-tumor, two different HCC cell lines (SMMC-7721, Hep-3B) and normal liver cell (L-02) were selected to examine the Gadd45β mRNA expression by PCR assay. As shown in Fig. 1e, Gadd45β mRNA level in all HCC cell lines was relatively lower than that in normal liver cell line. Similarity, western blot assay also showed that expression of Gadd45β protein was lower in HCC cell lines, when compared to normal liver cell line (Fig. 1f). From these results, we confirmed that expression of Gadd45β was significantly decreased in human liver tumor cells and tissues, when compared to normal liver cells and peri-tumor tissues.

Table 1 showed the 100 cases of patients’ demographics, clinicopathological factors, and the status of Gadd45β expression. Chi square test was used to examine the association of patients age, gender, hepatitis B, and Gadd45β expression, the result showed that there are no significant difference; However, there was a correlation between Gadd45β expression and HCC differentiation, this association was tested by Chi square (P < 0.05). The well differentiated HCC samples showed the higher expression of Gadd45β. The higher deficiencies of Gadd45β expression was observed in poorly differentiated samples. This suggested us that Gadd45β may play an important role in the stemness of liver cancer cells.

### Gadd45β expression inhibited the stemness properties of liver cancer cells

**Fig. 2:**
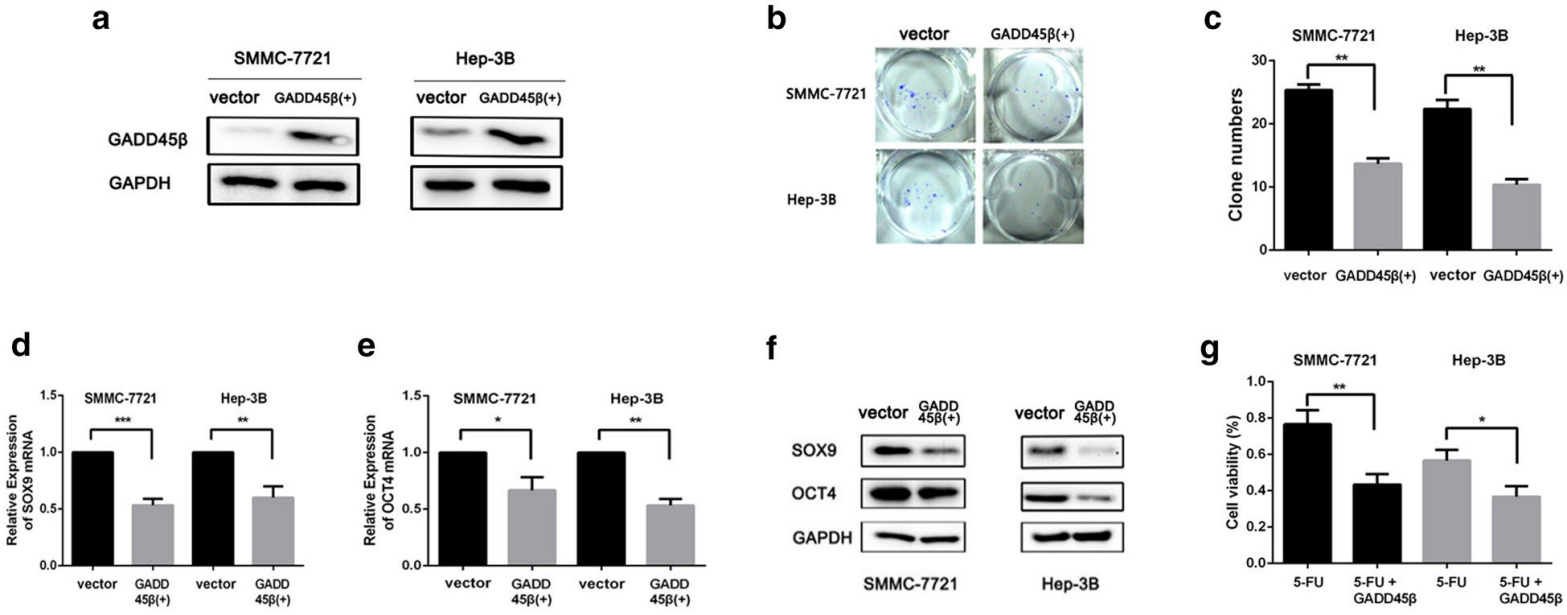
Gadd45β overexpression inhibited the stemness of liver cancer cells, enhancing apoptosis of cancer cells. **a** After the transfection of Gadd45β-expressing virus, western blot was performed to examine the Gadd45β expression. GAPDH was used as an internal reference. **b** The Gadd45β transfected SMMC-7721 and Hep-3B cells indicated the decreased colony formation. **c** Quantification of the clone numbers of cancer cells. Data are presented as the mean ± SD (** < 0.01). **d**, **e** PCR assay was used to detect the stemness makers’ expression in Gadd45β transfected cells. The results showed that transfection of Gadd45β resulted in the downregulation of SOX 9 and OCT4 mRNA expression in SMMC-7721 and Hep-3B cells. GAPDH was used as an internal reference (*P < 0.05; **P < 0.01; ***P < 0.001). **f** Western blot assay was then used to detect the level of SOX9 and OCT4 protein of cells. Gadd45β transfected SMMC-7721 and Hep-3B cells indicated a lower SOX9 and OCT4 protein level than vector control group. The protein levels were normalized by a comparison with the GAPDH level. **g** Cell viability assay showed that Gadd45β overexpression enhanced the cisplatin-induced apoptosis of SMMC-7721 and Hep-3B cells

To further determine the impact of Gadd45β on liver cancer cells, Gadd45β-expressing adenovirus were firstly transfected into SMMC-7721 and Hep3B cells. Western blot assay was performed to determine the Gadd45β expression (Fig. 2a). Then, the ability of colony formation in the transfected cells was examined. It was found that Gadd45β transfected SMMC-7721 and Hep-3B cells showed noticeable decrease in the colony formation, when compared with control group (**P < 0.01) (Fig. 2b, c), suggesting that the stemness of SMMC-7721 and Hep-3B cells may be inhibited by the overexpression of Gadd45β. Then, the expression of stem cell surface markers (SOX9 and OCT4) was assessed in Gadd45β transfected SMMC-7721 and Hep-3B cells, the results showed that SOX9 and OCT4 expression were reduced at both mRNA and protein levels in Gadd45β transfected liver cancer cells (*P < 0.05; **P < 0.01; ***P < 0.001) (Fig. 2d, e, f). Finally, to assess the effect of Gadd45β proteins on the apoptosis of liver cancer cells, cell viability assay was further used to assess the degree of apoptosis of cells with different treatment. The results showed that Gadd45β promoted the cisplatin induced apoptosis of SMMC-7721 and Hep-3B (*P < 0.05; **P < 0.01) (Fig. 2g). Therefore, we concluded that Gadd45β overexpression inhibited the stem cell properties of liver cancer cells, enhancing the chemosensitivity.

### The methylation of Gadd45β promoter in HCC tissues and liver cancer cell lines

**Fig. 3:**
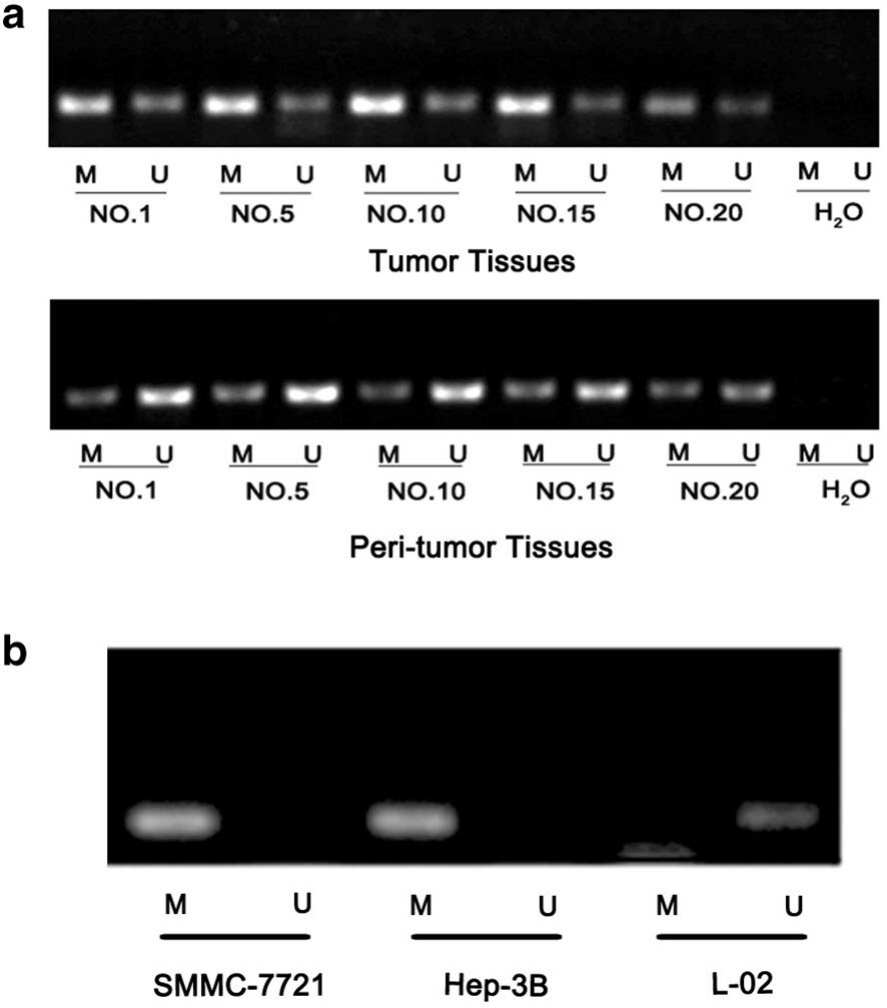
The methylation of Gadd45β promoter in HCC tissues and liver cancer cell lines. The status of methylation of Gadd45β promoter in HCC tissues and cells were studied by methylation-specific PCR (MSP) assay. **a** The HCC tumor tissues showed the obvious methylation status; however, the peri-tumor samples indicated strong unmethylated PCR products. **b** The determination of status of Gadd45β promoter methylation in liver cancer cell lines and normal liver cell. Methylated PCR products were detected in SMMC-7721 and Hep-3B cells; however, normal liver cells L-02 indicated unmethylated PCR products. “M” means methylation, “U” means un-methylation. The amplified methylation bands size is 137 bp; The unmethylated bands size is 128 bp. Negative control: pure water

It has been reported that methylation of promoter can silence CpG islands, and which was thought to be a epigenetic mechanism to inactive gene functions [18]. Furthermore, the study had shown that promoter methylation mediated the repression of Gadd45α and Gadd45β in gastric cardia adenocarcinoma [19]. We suspected that the silence of Gadd45β may be associated with the methylation of promoter in HCC. Thereby, the status of hypermethylation of CpG island was detected in HCC samples. The MSP primers were designed according to previous report [19]; the methylation-specific PCR (MSP) assay was performed in the selected clinical tumor specimens and surrounding peri-tumor tissues to qualitatively observe the methylated status of Gadd45β promotor. Among these five patients’ tumor samples, all PCR products could be amplified by the methylation-specific and unmethylated primers, but strong PCR products could be observed in the MSP system where the methylated primers were used. However, in peri-tumor samples, unmethylated primers were obviously amplified (Fig. 3a). Similarity, the normal liver cell L-02 showed an apparent un-methylation PCR products. Meanwhile, Gadd45β non-expressing HCC cell lines indicated the obvious methylation PCR products (Fig. 3b). The result suggested that there exists the obviously unmethylated Gadd45β in the liver peri-tumor tissues and normal cell, and where Gadd45β proteins were higher expressed; however, compared with peri-tumor tissues and normal liver cells, Gadd45β promotor were mainly methylated in tumor tissues and cancer cells, and where Gadd45β expression were mainly repressed. These results suggest that Gadd45β promoter methylation may influence the protein expression of Gadd45 gene in tumor tissues.

### Up-regulation of Gadd45β by pretreatment with 5-azacytidine increased sensitivity of liver cancer cells to cisplatin, and decreased the stemness of HCC cell lines

**Fig. 4:**
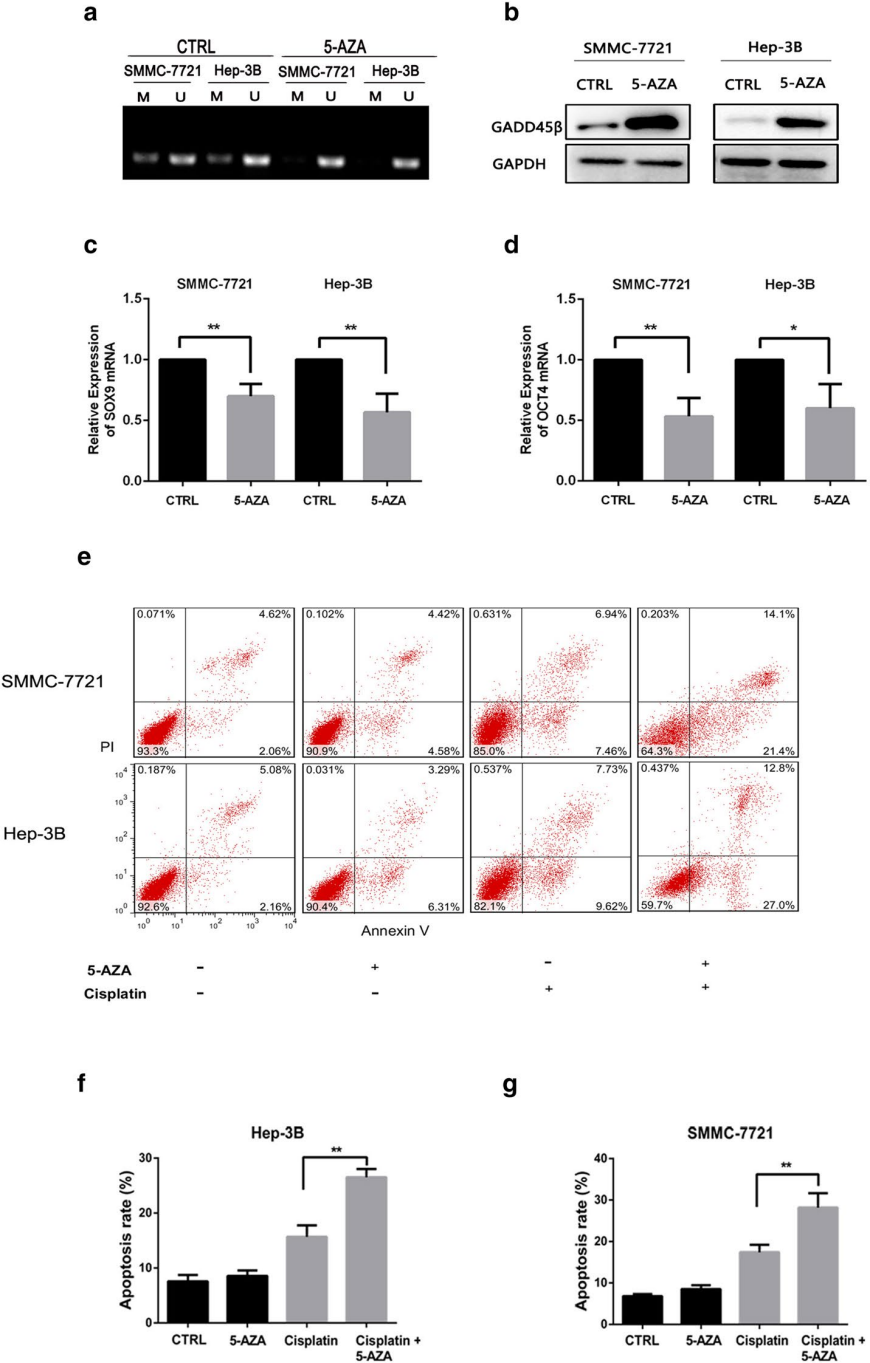
Up-regulation of Gadd45β by 5-azacytidine increased the chemosensitivity of liver cancer cells through decreasing the stemness of HCC cells. **a** After SMMC-7721 and Hep-3B cells were treated with 5-azacytidine, the methylation-specific PCR (MSP) assay showed that methylation of Gadd45β promoter was obviously inhibited. **b** Western blot assay confirmed that the cells treated with 5-azacytidine promoted the expression of Gadd45β protein. **c**, **d** RT PCR then indicated that 5-azacytidine inhibited the mRNA expression of SOX9 and OCT4 in SMMC-7721 and Hep-3B cells. GAPDH was used as an internal reference (*P < 0.05; **P < 0.01). **e** Flow cytometry was then used to detect the apoptosis of cells. the result showed that 5-azacytidine treatment significantly enhanced the cisplatin-induced the apoptosis of SMMC-7721 and Hep-3B cells than control group. **f**, **g** Quantification of the apoptosis of cancer cells. Data are presented as the mean ± SD (**P < 0.01)

5-azacytidine (5-AZA) is the DNA methyltransferase (DNMT) inhibitor, thus we hypothesized that induction of Gadd45β by pretreatment of 5-azacytidine would increase sensitivity of cancer cells to chemotherapy. Firstly, the depletion of Gadd45β methylation by 5-AZA was examined by MSP assay. The result showed that 5-AZA inhibited efficiently the Gadd45β methylation of SMMC-7721 and Hep-3B cells (Fig. 4a). And western blot confirmed that Gadd45β expression was unregulated in cells with treatment of 5-AZA (Fig. 4b). Furthermore, RT PCR assays showed that the 5-azacytidine also inhibited the SOX9 and OCT4 mRNA expression of HCC cell lines (Fig. 4c, d). Then the cells with or without the pretreatment of 5-AZA were treated with Cisplatin (2 μg/ml), flow cytometry showed that the increased apoptosis was observed in SMMC-7721 and Hep-3B cells with the pretreatment of 5-azacytidine, when compared to the cells without the pre-treament of 5-azacytidine (Fig. 4e). And quantitative analysis of apoptosis confirmed that these differences were significant (**P < 0.01) (Fig. 4f, g). Thereby, these results confirmed the combination of 5-azacytidine and cisplatin could promote the chemotherapy-mediated apoptosis in SMMC-7721 and Hep-3B cells by inhibiting the stemness of the liver cancer cells.

## Discussion

The tumor cellular response to chemotherapy still remain complex. Gadd45 proteins could rapidly response to genotoxic stress. However, the exact function of Gadd45 proteins in the response of tumor cells to genotoxic stress is still controversial. It was found that Gadd45α and Gadd45β proteins play a dual role in the apoptosis of tumor cells induced by genotoxic stress, showing both apoptotic and antiapoptotic function [20, 21]. In this study, the role of Gadd45β in the apoptosis of liver cancer cells was studied.

Firstly, the expression of Gadd45β in one hundred cases of HCC and surrounding peri-tumor tissues were studied. IHC results showed that the cancerous tissue displayed lower Gadd45β level than that in precancerous tissues. The results were further validated by PCR and western blot study. Furthermore, the tested liver cancer cell lines also showed a lower expression of Gadd45β, when compared to normal liver cell. And there exist an association between Gadd45β and HCC differentiation. Our observation keep consistent with many other studies [10, 22]. In order to study the impact of Gadd45β on liver cancer cells, Gadd45β-expressing adenovirus were transfected into SMMC-7721 and Hep-3B cells, we found that Gadd45β overexpression inhibited the stemness properties of cells, including the downregulation of SOX9 and OCT4 expression, reduction of colony formation. The stemness characters of cancer cells plays a critical role in the chemoresistance of cancer. Thus, we next examined the impact of Gadd45β on the chemotherapy-induced apoptosis in SMMC-7721 and Hep-3B cells, it was found that Gadd45β overexpression enhanced the cisplatin-induced apoptosis in SMMC-7721 and Hep-3B cells. Furthermore, it was found that methylation was involved in the downregulation of Gadd45β, and induction of Gadd45β by treatment of cells with DNA methyltransferase inhibitor (5-azacytidine) also increased the sensitivity of cancer cells to chemotherapy. Thus, Gadd45β may be a potential target for the therapy of hepatocellular carcinoma.

The exact mechanism of Gadd45 protein down-expression in cancer is still not clear. Up to now, the most studies in decreased expression of Gadd45 family is Gadd45a and Gadd45 g, and the main mechanism implicated in their decreased expression was due to the promoter methylation, such as many studies showed the frequent occurring of Gadd45a and Gadd45 g promoter methylation in many types of cancers [7, 14, 23, 24]. Besides the promoter methylation, the activated NF-κB also was suggested to result in the repression of Gadd45 a and Gadd45 g in cancers [25]. Moreover, because Gadd45a is the downstream mediator of p53, thereby many studies showed the p53 status was associated with expression of Gadd45a in many cancers [26, 27]. In case of Gadd45β, the promoter methylation is the most possible mechanism inducing the down-expression of Gadd45β. Besides, it was reported that the transforming growth factor (TGF)-B can induce Gadd45β expression [10]. In this study, it was found that Gadd45β methylation was correlated with their expression in HCC tissues and liver cancer cell lines.

## Conclusions

the expression of Gadd45β was repressed in HCC tissues and liver cancer cells, and the Gadd45β methylation may influence the expression. Furthermore, Gadd45β inhibited the stemness properties of liver cancer cells, enhancing the chemosensitivity of liver cancer cells. This finding help to further clarify the importance of Gadd45β in the progression of human HCC.

## Abbreviations

HCC: hepatocellular carcinoma
Gadd45β: growth arrest DNA damage-inducible gene 45β
IHC: immunohistochemistry
MSP: methylation-specific PCR
5-AZA: 5-azacytidine
